# Effects on asthma and respiratory allergy of Climate change and air pollution

**DOI:** 10.1186/s40248-015-0036-x

**Published:** 2015-12-22

**Authors:** Gennaro D’Amato, Carolina Vitale, Annamaria De Martino, Giovanni Viegi, Maurizia Lanza, Antonio Molino, Alessandro Sanduzzi, Alessandro Vatrella, Isabella Annesi-Maesano, Maria D’Amato

**Affiliations:** Division of Respiratory and Allergic Diseases, Department of Chest Diseases, High Speciality A.Cardarelli Hospital, Via Rione Sirignano,10, 80121 Naples, Italy; University “Federico II”, Medical School, Naples, Italy; Directorate General for Health Prevention, Ministry of Health, Rome, Italy; Institute of Biomedicine and Molecular Immunology and Institute of Clinical Physiology, National Research Council, Palermo and Pisa, Italy; First Division of Pneumology, High Speciality Hospital “V. Monaldi” and University “Federico II” Medical School, Naples, Italy; Second Division of Pneumology, High Speciality Hospital “V. Monaldi” and University “Federico II” Medical School, Naples, Italy; Department of Medicine and Surgery, University of Salerno, Salerno, Italy; Epidemiology of Allergic and Respiratory diseases department (EPAR), Institut Pierre Louis d’Epidémiologie et de Santé Publique (IPLESP UMRS 1136), Paris, France; Sorbonne Universités, UPMC Univ Paris 06, INSERM, Medical School Saint-Antoine, F75012 Paris, France

**Keywords:** Air pollution and asthma, Airway hypersensitivity, Allergic airway diseases, Climate changes, GARD, Pollen allergy: Thunderstorm asthma, Weather and asthma

## Abstract

The major changes to our world are those involving the atmosphere and the climate, including global warming induced by anthropogenic factors, with impact on the biosphere and human environment. Studies on the effects of climate changes on respiratory allergy are still lacking and current knowledge is provided by epidemiological and experimental studies on the relationship between allergic respiratory diseases, asthma and environmental factors, like meteorological variables, airborne allergens and air pollution.

Epidemiologic studies have demonstrated that urbanization, high levels of vehicle emissions and westernized lifestyle are correlated with an increased frequency of respiratory allergy, mainly in people who live in urban areas in comparison with people living in rural areas.

However, it is not easy to evaluate the impact of climate changes and air pollution on the prevalence of asthma in general and on the timing of asthma exacerbations, although the global rise in asthma prevalence and severity could be also considered an effect of air pollution and climate changes. Since airborne allergens and air pollutants are frequently increased contemporaneously in the atmosphere, enhanced IgE-mediated response to aeroallergens and enhanced airway inflammation could account for the increasing frequency of respiratory allergy and asthma in atopic subjects in the last five decades. Pollen allergy is frequently used to study the interrelationship between air pollution and respiratory allergic diseases such as rhinitis and bronchial asthma. Climatic factors (temperature, wind speed, humidity, thunderstorms, etc) can affect both components (biological and chemical) of this interaction. Scientific societies should be involved in advocacy activities, such as those realized by the Global Alliance against chronic Respiratory Diseases (GARD).

## Review

The global environment of Earth is facing deep changes: many of these can affect respiratory health and increase asthma frequency in the general population. In the last century the massive increase in emissions of air pollutants due to the economic growth has made air quality a major problem for many of industrialized countries, and an emerging problem for the rest of the world. Increased concentrations of greenhouse gases, and especially CO_2_, in the earth’s atmosphere have already substantially warmed up the planet, affecting biosphere and biodiversity and determining more severe and prolonged heat waves, temperature variability, air pollution, forest fires, droughts, and floods: such factors put respiratory health at risk [1–7]. These changes in climate and air quality have a quantifiable impact for both morbidity and mortality of asthma and other respiratory diseases.

Current projections suggest that the world population will rise to 9 billion by 2050. There are 20 cities populated by more 10 million inhabitants and two thirds of mankind are expected to live in megalopolis by 2020 [6]. It is to point out that in the last fifty years 50 % of pluvial forests on our planet have been destroyed, and each year 13 million hectares of forest are being destroyed or deteriorated.

Food cultivation on wasted areas of tropical pluvial forests induced about 35 % of deforestation in countries in South America, 70 % in Africa and 50 % in Asia [6].

Desert sand, sea salt, wild fires and volcanic ash are components of natural pollution which are added to man-made particulates that pollute the air. The spread of wood for heating observed in the last decades is another important source of pollution. Although wood energy production is associated with lower greenhouse gas emissions, it releases many other gaseous and particulate pollutants [6].

Each year hundreds of millions of tons of waste are generated from homes and businesses. The problem is aggravated by a model of development based on the intensive use of fossil fuels, which is the main component of the worldwide energy production system. Another determinant has been the increased soil use, mainly the deforestation for agricultural purposes [6].

A recent position statement on climate change and health impacts issued by the European Respiratory Society (ERS) was developed after a workshop co-organized by the HENVINET Project and the American Thoracic Society [8]. The position statement highlights climate related health impacts, including deaths and acute morbidity due to heat waves; increased frequency of acute cardio-respiratory events due to higher concentrations of ground level ozone; changes in the frequency of respiratory diseases due to trans-boundary particle pollution; altered spatial and temporal distribution of allergens (pollens, moulds and mites) and some infectious disease vectors. Such impacts will not only affect individuals with existing respiratory diseases but will likely increase the incidence and prevalence of respiratory conditions [8]. The 2015 WHO European Region document [3] estimated economic costs for 2010: “The annual economic cost of premature deaths from air pollution across the countries on the WHO European region stood at US $ 1.431 trillion and the overall annual economic cost of health impacts and mortality from air pollution, including estimates for morbidity costs, stood at US$ 1.575 trillion.”

### Extreme temperature events

According to the Fifth Assessment Report (AR5) of the Intergovernmental Panel on Climate Change (IPCC) [9] the average global temperature in the period 2003-2012 was 0.78 °C higher than in the second half of 1900 century. Extreme heat is one of the most important global causes of weather-related mortality: more frequent and intense heat waves are determined by climate change. Heat and heat waves are projected to increase in severity and frequency with increasing global mean temperatures.

The effect of heat waves on mortality is well documented [10]. It has been observed a rapid rise in the number of hot days and severe meteorological events [10–13] associated with excess deaths across Europe: the 2003 and 2012 heat waves temperatures reached or went beyond 35 °C degrees [12]. In Europe, over 70.000 excess deaths were observed in 12 European countries in the heat wave summer of 2003. Summer temperatures as high as those are expected to be the norm by the middle of the century in Europe [12]. There is evidence of an increased number of deaths and acute morbidity especially among respiratory patients due to heat waves. For each degree Celsius rise in temperature, the risk of premature death among respiratory patients is up to six times higher than in the rest of the population [5]. The increase in respiratory mortality (relative risk) is larger than the increasing total or cardiovascular mortality [14]. Moreover, climate scenarios for the next century predict that the warming will be associated with more frequent and more intense heat waves in wide areas of our planet with increased risk of wildfires and desertification. Heat and drought conditions contribute to wildfire risks. Smoke emissions can travel hundreds of kilometers downwind of fire areas, exposing people to a complex mixture of fine particles, ozone precursors, and other health-harming compounds [15, 16]. One recent worldwide estimate is that 339,000 deaths annually may be attributable to landscape fire smoke [16]. Respiratory and cardiovascular hospital admissions and emergency room visits increase in response to wildfire smoke exposures, strongly associated with PM levels [16, 17]. Drought conditions create multiple health challenges: in dry conditions, more pollen, dust, particulates, and when present, wildfire smoke which can irritate respiratory epithelium, exacerbate chronic respiratory illnesses, asthma, and increase risks for acute respiratory infection [18, 19] (Table 1).

**Table 1:** Effects of climate change on respiratory diseases

An increased number of deaths and acute morbidity especially among respiratory patients due to heat waves
An increased frequency of cardio-respiratory attacks due to higher concentrations of ground-level ozone
Changes in the frequency of respiratory disease due to transboundary long-range air pollution
Altered distribution of allergens and some infectious disease vectors

### Outdoor and indoor air pollution

Air pollution is an alteration of air quality due to natural or anthropogenic emissions of physical, chemical and biological substances. It’s the environmental factor with the largest impact on respiratory health in Europe [1, 2]. The lung is one of the major sites of interaction with environmental particulates. PM consists of three fractions: a coarse fraction, PM_10-2,5_ (2,5-10 μm); a fine fraction PM_2,5-0,1_ (2,5-0,1 μm); and ultrafine nanosize fraction, PM_0,1_ (≤0,1 μm). The coarse fraction is considered responsible for hazards in the larger airways, whereas PM _2,5_ reaches the gas ex-change region. However only the smallest fraction, the nanosize particles, could penetrate in the alveoli through the air-blood tissue barrier into the capillaries, after being deposited on the alveolar wall. Besides entering tissues and cells in the lung, facilitated by the minute size, these particles may translocate through the blood circulation to all other organs [20–22]. Therefore, it is this fraction of PM that should be given particular attention as far as adverse health effects are concerned.

Air pollutants cause direct cellular injury or induce intracellular signaling pathways and transcription factors that are known to be sensitive to the oxidative stress [23–28]. Air pollution has been related to asthma. Outdoor air pollution exacerbates asthma in those patients who already have the condition [29]. Outdoor levels of air pollutants have been associated with asthma incidence and prevalence (“the urban factor”) at the population level.

Particle pollution, vehicle exhaust and ground level ozone are the most important types of hazardous pollutants. Ozone is a powerful oxidant that has been associated with persistent structural airway and lung tissue damage, as well as with more severe asthma symptoms and increase in respiratory hospital admissions and deaths in Europe and the USA [27–29]. It is estimated that there will be 1,500 more annual ozone associated deaths by the year 2020 in the UK alone [28]. Pollution models for climate change scenarios predict an increase in ozone concentrations over large areas, while the effect on particle concentrations is less clear [27]. The short-term effects of ozone on daily mortality and respiratory disease are extensively studied, while there is only limited documentation of long-term effects on mortality [27, 28].

Climate factors like temperature, wind, and precipitation play important roles in determining patterns and concentrations of air pollution over multiple scales in time and space. They operate through changes in air pollution emissions, transport, dilution, chemical transformation and deposition of air pollutants (particulate and gaseous like in particular secondary pollutants like ozone).

Today, there are tens of thousands of known or suspected air pollutants, sometimes acting in synergy each other as well as with other parameters (temperature, wind, etc). However, very few of them are monitored; only those covered by regulations. Within the premises, it can be found a cocktail of toxic chemical pollutants and allergens: paints, adhesives, flooring, cleaning products, dye waste, heaters and gas cooking, as well as other toxic compounds, such as dust from lead paint, radon and asbestos.

Other forms of air pollution are represented by the excess of pollens and moulds. In addition, people are exposed to air pollution also within the premises such as houses, offices, schools where they spend between 80 and 90 % of their time, with its 3,000 compounds identified to date and its 5 billion particles per cigarette, tobacco smoke is unquestionably the most formidable air pollutants related to human activity [6].

Positive associations have been observed between urban air pollution and respiratory symptoms in children, and the literature contains many reports of a relation between motor vehicle exhausts and acute or chronic respiratory symptoms in children living near traffic [30–37].

Air pollution can negatively influence lung development in children and adolescents [30–37]. Most studies suggested the adverse effects of air pollution on children’s lung function and respiratory symptoms. Deficits in lung function were associated with a correlated set of pollutants that included nitrogen dioxide, acid vapor, fine particulate matter (PM_2,5_), and elemental carbon. Deficits in lung function during young adulthood may increase the risk of respiratory conditions, such as episodic wheezing that occurs during a viral infection. However, the largest effects of pollution related deficits might occur later in life, since reduced lung function is a strong risk factor for complications and death during adulthood [38–41]. Moreover, PM can affect also the cardiovascular system. According to the British Heart Foundation, airborne particulate matter (PM) can increase the risk of CVD in three ways [42]. Firstly, PM can stimulate receptors in the lungs that disrupt the nervous system and cause changes to heart rhythm. Secondly, inhaled particles can cause inflammation of the lungs and then damage the cardiovascular system as inflammatory chemicals pass into the blood. At last, ultrafine particles may pass into the blood and directly affect blood vessels [42].

Prediction about the effects of climate change on health-related air pollution is hampered by several limits: future emissions depend on numerous factors, such as population growth, economic development, energy use and production; current knowledge about weather effects on air pollution is still unsatisfactory; there is still a need of better emission inventories and observational datasets. As regards indoor pollution, indoor levels of air pollutants other than environmental tobacco smoking have also been related to asthma prevalence or symptoms by some studies [43–45]. Consistent results support short-term (aggravation) and also long-term (prevalence augmentation) effects on asthma of poor air in indoor settings [43–45]. Environmental tobacco smoke is one of the most important risks for respiratory symptoms and diseases worldwide. The evidence is also reliable for indoor nitrogen dioxide and particulate matter, which have been associated with asthma. Whereas formaldehyde and volatile organic compounds seem to be important pollutants in indoor settings, there are still few papers on asthma and bronchitis.

Moulds have been associated with increased risks of asthma and COPD.

### Allergens and allergic responses

The effects of climate change on respiratory allergy are still under-studied. Allergic respiratory diseases and asthma are a result of environmental and immunologic interaction. As a consequence of the increased evidence of the link between climate change and asthma and allergy, it is important to focus on the underlying factors: global warming is expected to affect the start, duration, and intensity of the pollen season on the one hand, and the rate of asthma exacerbations due to respiratory infections on the other [6, 44–47]. Data provided by 30 years of observations within the International Phenological Gardens network showed that spring events advanced by six days, the highest rate of phenological changes being observed in Western Europe and Baltic regions. Conversely, phenological trends appear to be different in Eastern border of Europe, sometimes showing a 1-2 weeks later start of the phases. Due to the earlier onset, the pollen seasons are more often interrupted by adverse weather conditions in late winter/early spring [44–47]. Duration of the pollen season is also extended, especially in summer and in late flowering species. Acute (short term) exposure to particulate air pollution has been found to be associated with increases in the rates of daily asthma attacks, hospital admissions, and mortality. In addition to lung damage epidemiological and toxicological studies have shown adverse effects of PM on the hearth.

The climate changes projected during the next century will influence plant and fungal reproductive systems and alter the timing, production, and distribution of aeroallergens. Increased exposure to allergens as a result of global warming, combined with exposure to pollutants that act synergistically to intensify the allergic response, could point to increased respiratory problems in the future [6]. In fact, climate change is likely to influence vegetation, with consequent changes in growth and reproductive cycles and in the production of allergenic pollen (seasonal period and intensity). In addition, weed species are expected to proliferate. These changes can vary from one region to another, since some areas receive greater amounts of UV radiation and/or rainfall than others (Table 2).

**Table 2:** Why climate change is correlated with pollen allergy?

Increase and faster plant growth
Increase in the amount of pollen produced by each plant
Increase in the amount of allergenic proteins contained in pollen
Increase in the start time of plant growth and therefore the start of pollen production and earlier and longer pollen seasons

### Thunderstorm asthma

An increasing body of evidence shows the occurrence of severe asthma epidemics during thunderstorm in the pollen season, several epidemics of asthma have been reported following thunderstorms in various geographical zones, mainly in Europe and Australia [48, 49]. The asthma epidemics related to thunderstorms are limited to seasons when there are high atmospheric concentrations of airborne allergenic pollens (subjects with pollen allergy who stay indoors with the window closed during thunderstorms are not involved); further, there are no observations on the involvement of asthma in non-allergic subjects. Much remains to be discovered about the relationship between asthma attacks and thunderstorms, but there are reasonable evidences in favor of a causal link between them in patients suffering from pollen allergy. In particular, during the first phase of a thunderstorm, that is the first 20-30 min, there is evidence of high respirable allergen loads in the air. This is due to dry updrafts that, during a thunderstorm, entrain whole pollens into the high humidity at the cloud base where pollens may rupture and cold downdrafts carry pollen fragments (pollen grains are too large to penetrate the deeper airways) to ground level where outflows distribute them. Due to strong electric fields that develop during thunderstorms, positive ions are released from the ground and could attach to particles and/or electric charge may enhance pollen rupture, thus, inducing bronchial hyper-responsiveness [48, 49]. As a consequence, subjects affected by pollen allergy, not only asthmatic patients but also subjects affected by seasonal rhinitis without asthma symptoms, should be alert to the danger of being outdoors during a thunderstorm in the pollen season, as such events may be an important cause of severe exacerbations (Table 3).

**Table 3:** The evidence about thunderstorm related epidemics of rhinitis and asthma exacerbations

The occurrence of epidemics is strictly linked to thunderstorm
The epidemics related to thunderstorm are limited to late spring and summer when there are high levels of airborne pollen grain
There is a close temporal association between the arrival of the thunderstorm, a major rise in the concentration of pollen grains and the onset of outbreak
Subjects with pollen allergy who stay indoors with window closed during thunderstorm, are not involved
There is a major risk of severe asthma for subjects who are not under antiasthma correct treatment, but subjects with allergic rhinitis and without previous asthma can experience severe bronchoconstriction.

### The Global Alliance against chronic Respiratory Disease (GARD)

In view of these reviewed evidences, it is important that the scientific community engages in advocacy activities. An important tool for pulmonologists and allegologists is GARD, a partnership among WHO, governments, scientific societies and patients organization, launched in Beijing in 2006.

Hundreds of millions of people of all ages suffer from chronic respiratory diseases which include asthma and respiratory allergies, chronic obstructive pulmonary disease, occupational lung diseases and pulmonary hypertension. More than 500 million patients live in developing countries or in deprived populations. Chronic respiratory diseases are increasing in prevalence. Although the cost of inaction is clear and unacceptable, chronic respiratory diseases and their risk factors receive insufficient attention from the healthcare community, government officials, media, patients and families. The Fifty-Third World Health Assembly recognised the enormous human suffering caused by chronic diseases and requested the World Health Organization (WHO) Director General to give priority to the prevention and control of chronic diseases, with special emphasis on developing countries. This led to the formation of the WHO Global Alliance against Chronic Respiratory Diseases (GARD). GARD is a voluntary alliance of organisations, institutions and agencies working towards a common vision to improve global lung health according to local needs. GARD is developed in a stepwise approach using the following three planning steps: estimate population need and advocate action; formulate and adopt policy; and identify policy implementation steps [50, 51].

The 2008-2013 WHO action plan on noncommunicable diseases (NCDs) includes chronic respiratory diseases as one of its four priorities. Major chronic respiratory diseases (CRDs) include asthma and rhinitis, chronic obstructive pulmonary disease, occupational lung diseases, sleep-disordered breathing, pulmonary hypertension, bronchiectiasis and pulmonary interstitial diseases. A billion people suffer from chronic respiratory diseases, the majority being in developing countries. CRDs have major adverse effects on the life and disability of patients. Effective intervention plans can prevent and control CRDs, thus reducing morbidity and mortality. A prioritised research agenda should encapsulate all of these considerations in the frame of the global fight against NCDs. This requires both CRD-targeted interventions and transverse NCD programmes which include CRDs, with emphasis on health promotion and disease prevention [52].

The prevention of CRDs and reducing their social and individual impacts means modifying environmental and social factors and improving diagnosis and treatment. Prevention of risk factors (tobacco smoke, allergens, occupational agents, indoor/outdoor air pollution) will significantly impact on morbidity and mortality. The Italian Ministry of Health (MoH) has made respiratory disease prevention a top priority and is implementing a comprehensive strategy with policies against tobacco smoking, indoor/outdoor pollution, obesity, and communicable diseases. Presently these actions are not well coordinated. The Global Alliance against Chronic Respiratory Diseases (GARD), set up by the World Health Organization, envisages national bodies; the GARD initiative in Italy, launched 11/6/2009, represents a great opportunity for the MoH. Its main objective is to promote the development of a coordinated CRD program in Italy. Effective prevention implies setting up a health policy with the support of healthcare professionals and citizen associations at national, regional, and district levels. What is required is a true inter-institutional synergy: respiratory diseases prevention cannot and should not be the responsibility of doctors alone, but must involve politicians/policymakers, as well as the media, local institutions, and schools, etc. GARD could be a significant experience and a great opportunity for Italy to share the GARD vision of a world where all people can breathe freely [53].

GARD Italy has been engaged in the following projects:

- A) Period 2010-2012:

1. Prevention program for schools of indoor risks for respiratory and allergic diseases
2. Smoking and environment in the household
3. Development of predictive medicine within respiratory diseases
4. Early diagnosis implementation through pathways for health staff
5. Care continuity
  B) Period 2012-2014:
  1. Environment and respiratory diseases
  2. Surveillance of respiratory diseases
  3. Education: asthma and allergies in childhood
  4. Smoking and environment in the household
  5. Care continuity: respiratory impairment
    Within the Program “Environment and respiratory diseases”, a national Workshop, convened by Annamaria De Martino, Italian MoH officer, at the Ministry of Health building in Rome has been held on December 16-17, 2013, to contribute to the European “Year of the Air”. The topics have been: Climate, Air Quality and Respiratory Health. Italian and European projects (EPIAIR, REVIHAP, ESCAPE, OFFICAIR, for outdoor pollution; SEARCH, HESE, HESEINT, SINPHONIE, for indoor pollution) have been reviewed. The opinions of patients have been described by the representatives of asthma and COPD patients associations. A round table on “Environment and Health: The role of the Institutions” has been performed by representatives of the MoH, Ministry of Environment, Ministry of Education, University and Research, Regions, National Health Institute, National Environmental Protection Institute
    During the Italian Presidency of the Council of European Union (July 1- December 31, 2014), a Meeting of Chief Medical, Chief Nursing and Chief Dental Officers at the Ministry of Health building in Rome has been held on October 6-7, 2014. Giovanni Viegi (Italian representative in the international GARD) has delivered a speech on “Prevention and Organizational Models for Respiratory Chronic Diseases (including Allergies) and has collaborated with Giselda Scalera and Giovanna Laurendi, Italian MoH medical officers, to draft a working document on “CRD: an important public health problem”, with the following sections: Definition; Epidemiology; EU and international scenario; Proposal for discussion (CRD surveillance; CRD care) aimed at EU strategy for prioritizing CRD prevention, diagnosis and care.
    At last, convened by Annamaria De Martino, Italian MoH medical officer, a Workshop on “Indoor Air Pollution and Health in Italian Schools” at the Ministry of Health building in Rome has been held on February 23, 2015. In such occasion, results of the Italian CCM funded project on “Exposure to indoor pollutants: guidelines for the assessment of risk factors in school environment and definition of the measures for respiratory health protection of schoolchildren and adolescents”, coordinated by ISS and CNR, have been reported.

## Conclusions

Health effects of climate change include an increase in the prevalence of allergic respiratory diseases, exacerbations of chronic obstructive lung disease, premature mortality, and declines in lung function [6, 54, 55]. Climate change, mediated by greenhouse gases, causes adverse health effects to the most vulnerable patient populations such as elderly, children, patients with chronic non-communicable diseases and those in distressed socioeconomic strata.

Considering these aspects governments worldwide and international organizations such as the World Health Organization and European Union are facing a growing problem of the respiratory effects induced by gaseous and particulate pollutants arising from motor vehicle emissions.

In addition, climate change may significantly worsen health inequities within and among countries and put additional stress on poorer groups.

In conclusion, strategies to reduce climate changes and air pollution are political in nature, but citizen and in particular health professionals and societies must raise their voices in the decision process to give strong support for clean policies on both national and international levels.

Policy changes are beginning to impact greenhouse gas production in many parts of the world.

These efforts are crucial for reducing future impacts, but because over-all global emissions continue to rise, adaptation to the impacts of future climate variability will also be required.

An important advocay tool is the last encyclics letter of Pope Francis: *“*There is an urgent need to develop policies so that, in the next few years, the emission of carbon dioxide and other highly polluting gases can be drastically reduced, for example, substituting for fossil fuels and developing sources of renewable energy” [56].
